# On the Application of Replica Molding Technology for the Indirect Measurement of Surface and Geometry of Micromilled Components

**DOI:** 10.3390/mi8060195

**Published:** 2017-06-21

**Authors:** Federico Baruffi, Paolo Parenti, Francesco Cacciatore, Massimiliano Annoni, Guido Tosello

**Affiliations:** 1 Department of Mechanical Engineering, Technical University of Denmark, Produktionstorvet, Building 427A, 2800 Kgs. Lyngby, Denmark; guto@mek.dtu.dk; 2 Department of Mechanical Engineering, Politecnico di Milano, via la Masa 1, 20100 Milan, Italy; paolo.parenti@polimi.it (P.P.); francesco.cacciatore@polimi.it (F.C.); massimiliano.annoni@polimi.it (M.A.)

**Keywords:** replica technology, roughness, micromilling, surface metrology, dimensional micro metrology

## Abstract

The evaluation of micromilled parts quality requires detailed assessments of both geometry and surface topography. However, in many cases, the reduced accessibility caused by the complex geometry of the part makes it impossible to perform direct measurements. This problem can be solved by adopting the replica molding technology. The method consists of obtaining a replica of the feature that is inaccessible for standard measurement devices and performing its indirect measurement. This paper examines the performance of a commercial replication media applied to the indirect measurement of micromilled components. Two specifically designed micromilled benchmark samples were used to assess the accuracy in replicating both surface texture and geometry. A 3D confocal microscope and a focus variation instrument were employed and the associated uncertainties were evaluated. The replication method proved to be suitable for characterizing micromilled surface texture even though an average overestimation in the nano-metric level of the *Sa* parameter was observed. On the other hand, the replicated geometry generally underestimated that of the master, often leading to a different measurement output considering the micrometric uncertainty.

## 1. Introduction

In recent decades, the demand for micro components has steadily increased in many engineering sectors such as biotechnology, avionics, medicine, automotive, etc. [1,2]. With the aim of meeting the new challenging requirements in terms of precision and accuracy, the world of manufacturing reacted either by developing brand new technologies or by downscaling well-established ones. Micromilling belongs to the second class of processes, being the miniaturized adaptation of conventional milling technology. The ability to produce full three-dimensional micro components with relatively high material removal rate makes this process suitable for producing molds and inserts employed in replication techniques such as micro injection molding [3]. When evaluating the quality of a machined mold, two main characteristics must be addressed, namely the geometrical accuracy and the surface topography. In fact, mold dimensional features and surface topographies are directly transferred to replicated products [4]. Furthermore, the mold surface can directly affect the replication process since it influences, for instance, demolding forces in micro injection molding [5]. A comprehensive and detailed knowledge of micromilled mold surfaces and geometries is therefore necessary for optimizing the product performance.

When dealing with micromilling, numerous phenomena, negligible for conventional milling, become significant. One of them is related to the minimum chip thickness effect [6]. When machining with chip thickness values comparable to the tool edge radius, effects such as plowing and elastic recovery become dominant, causing a decrease of dimensional accuracy and an increase of surface roughness, in particular for soft-state metallic alloys [7].

At present, many dimensional and topographical measurement solutions can be applied to micro components [8]. Among all of them, optical instruments are strongly emerging [9] because of their peculiar advantages. Firstly, their contact-less and non-destructive nature makes them suitable for measuring soft and very small components: even a small force would in fact invalidate the measurement or damage the sample. Furthermore, most optical measuring techniques allow an areal surface characterization, which has more statistical significance in comparison to a profile one [10]. Finally, they are potentially faster than typical contact instruments such as coordinate-measuring machines (CMMs) and stylus profilometers. However, certain features, for instance micro holes or micro cavities, are inaccessible for optical lenses, since an insufficient amount of light is reflected back to the measuring objective. In other cases, samples may not physically fit under the microscope. Inaccessible features are typical in micro molds for injection molding applications, where high aspect ratio features must often be replicated [11,12,13]. In such cases, new solutions must be employed to perform dimensional and surface measurements without adopting destructive inspections. Among them, replica molding technology is the most promising one. This method is based on the replication of inaccessible features by means of casting-like soft polymeric media such as polydimethylsiloxane (PDMS) or two-component silicone polymers [4]. The common replication procedure simply consists in casting the replication media over the component to be replicated. After the curing is completed, the replica is removed and then measured, providing and indirect quantitative information of the actual geometry and surface. The use of such method is well-established in medical and dental fields [14,15] where specimens simply cannot be placed under a measuring instrument. It is also widely employed in non-destructive metallographic analysis of mechanical components [16,17]. For dimensional and surface metrology tasks, replication kits based on a two-component polymer are nowadays widely used [18,19]. Silicone based materials are usually employed, since they ensure almost no shrinkage over a wide range of curing temperature. When using these kits, the polymer and the curing agent are automatically mixed in a disposable nozzle. A dispensing gun is utilized for directing the casting of the replication media. Tosello et al. [20] successively employed this method to monitor the tool wear of a mold for Fresnel lenses production presenting micro structures with a height of 23 µm.

In recent decades, few research papers investigated the replication performance of fast replication media used in indirect metrology tasks. Madsen et al. [21] studied the shrinkage of a PDMS material utilized for measuring nano-metric geometries. The authors considered several mixing ratios and curing temperature levels and investigated the repeatability of the process through repeated replicas. The authors concluded that the utilized PDMS shrinks linearly between 1% and 3 %, leading to dimensions that are smaller than the ones of the replicated master. In a recent paper, Goodall et al. [22] analyzed the accuracy and precision of seven different silicone based replication media utilized for quantitative surface texture characterization. Two masters, one smooth and one rough lower jaw teeth, were replicated. Most of the areal surface texture parameters and fractal parameterization were employed to carry out comparisons. It was found that low viscosity media generally achieve higher accuracy and precision. However, the authors highlighted a need for standardization, since results from different impression media were not comparable. Finally, Gasparin et al. [23] investigated the surface texture replication performance of three replication media (one hard and two soft). Two calibrated roughness standards were used as masters. Measurements were performed with optical and tactile instruments. A replication degree up to 96% was reached, proving that the measured deviations fell inside the uncertainty range. Since the nominal *Ra* value of the measured standard was 500 nm, the authors’ considerations cannot be directly extended to micromilled surfaces, which are usually smoother (in most applications, *Ra* ranges between 50 nm and 250 nm [24,25,26,27] on both flat and free-form parts).

Despite several approaches have been proposed in literature [28,29], the use of surface topography prediction models in industrial micromilling applications is still extremely limited. This is due to the lack of robustness that the models show with respect to the variability of the material characteristics, tool micro-geometry and tool wear. Therefore, the measurement of micromilled surface after machining represents the standard practice that machinists still adopt for verifying the cutting process results. The replication and the following indirect measurement are thus the preferable solution for characterizing the surface topography of inaccessible micromilled features.

At present, the literature still lacks a study focused on the performance verification of a replica procedure specifically applied to micromilled components.

The aim of this paper is to evaluate the performance of a commercial silicone replication media (RepliSet [19], Struers^®^, Ballerup, Denmark) in the indirect measurement of micromilled surfaces and geometries made from mold steel. In order to accomplish this, a quantitative comparison between micromilled masters and their replicas was carried out. In particular, the performance in replicating both surface topography and geometry was assessed separately using two different benchmark samples.

## 2. Materials and Methods

In order to assess the surface replication fidelity of the silicone media, two different samples were designed and then machined.

The first one was used to assess the replication performance related to indirect surface roughness measurements. Different benchmark surfaces were machined and then replicated. Micro-roughed and micro-finished surfaces were obtained by varying the radial depth of cut. Two materials were milled: AISI 440 hardened (AISI 440 H, hardness = 60 HRC) and AISI 440 annealed (AISI 440 A, hardness = 18 HRC). These two stainless steels represent a suitable choice for mold manufacturing and are expected to produce different surface topographies in relation to the specific material characteristics, as for instance hardness, grain size and specific cutting force. In particular, being AISI 440 A in a soft state, the risk of plowing is certainly higher than for the harder AISI 440 H [7].

The second sample was used to investigate the replication capability related to indirect geometrical measurements. Five micromilled pockets were produced on the same two aforementioned materials and successively replicated. The geometry of the pockets was varied in order to assess the replication fidelity of the silicone media at different penetration depths.

### 2.1. Micromilled Surfaces

The consideration of surface topography is the first step for the assessment of any replication method. In micromilling surface generation, several factors affect the final surface topography. In particular, milling process parameters, milling strategy and the geometry of the adopted tool all play an important role. In this study, three typologies of micromilled surface were produced on both the materials. A coated WC Round End Mill (Mitsubishi Materials Corporation, Tokyo, Japan) (2 flutes, diameter = 1 mm, Corner Radius = 0.1 mm and cutting edge radius = 6 µm) was used for the tests on an ultra-high precision KERN Evo micromilling machine. Table 1 shows the cutting parameters for the three machined surfaces.

**Figure 1:**
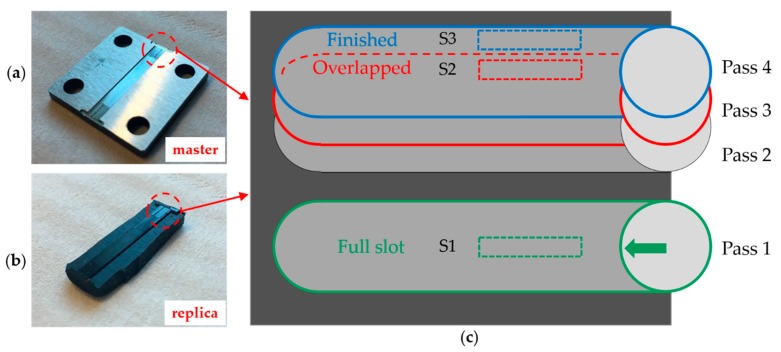
Micromilled surfaces and their replicas: (**a**) AISI 440 H sample. The two replicates of the machined surfaces are visible. (**b**) Silicone replica of the AISI 440 H sample. (**c**) Scheme of the micromilled surfaces. The four mill passes are presented in their machining sequence. The three measured surfaces S1, S2 and S3 are indicated with a dashed line. The green arrow shows the milling direction.

The first surface, designated S1, was machined in full slot (i.e., with radial depth of cut *a*_e_ equal to 100% of the mill diameter *D*_C_). S2 and S3 were instead obtained by imposing a 30% radial depth of cut. S2 designates the overlapped surface area, generated by two subsequent tool passes. S3 is generated by the last mill pass. Figure 1 shows a scheme of the surface design. These specific surfaces represent typical conditions of a mold manufacturing process: S1 conditions are distinctive of roughing operations (e.g., during pocket milling), where full slot machining is employed to minimize the machining time. S2 and S3 are representative of finishing operation, where the final surface topography and appearance are generated using a lower value of the radial depth of cut. S1 surface roughness is therefore expected to be higher than for S2 and S3.

The other milling parameters were kept constant in the three cases. The feed per tooth *f*_z_ was set at the lowest limit for the selected mill. The axial depth of cut *a*_p_ was limited to 50 µm for all the tests to avoid the onset of chatter conditions which can lead to uncontrolled and defective surface generation. In order to incorporate the effect of the tool wear in the surface generation, the same tool was used for machining all the samples.

Two replicates for each surface type, designated as Sample 1 and Sample 2, were generated on the same metal workpiece (see Figure 1a). Six surfaces per material were therefore available for replication.

### 2.2. Micromilled Geometrical Features

In order to assess the performance of the replication media in terms of micromilled geometry replication, five micromilled pockets were produced on the two materials. Such features represent a generalization of two-dimensional channels and cavities that are often present in micro molds. For carrying out the machining, a WC flat end mill (Sandvik Coromant, Sandviken, Sweden) (2 flutes, diameter = 0.5 mm and cutting edge radius = 6 µm) was used on the same micromilling machine. The cutting parameters (see Table 2) were kept constant for all the machined pockets. Once again, the same tool was utilized for producing all the samples.

**Figure 2:**
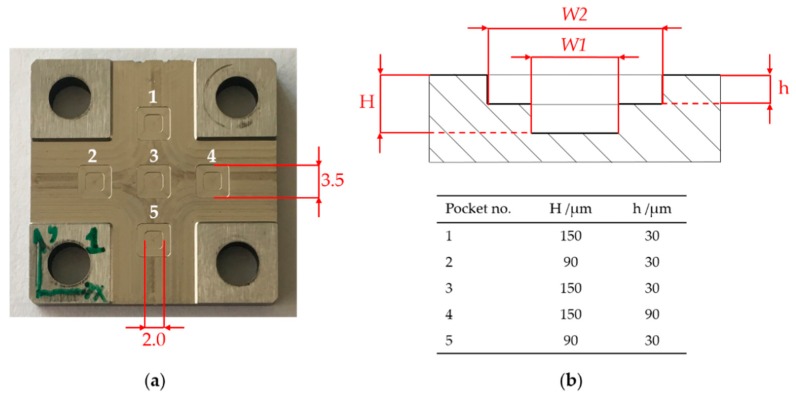
(**a**) Micromilled pockets with nominal width dimensions; and (**b**) pocket depths and the two measurands *W1* and *W2*.

Figure 2 shows the geometrical characteristics of the micromilled pockets. In particular, the five two-stepped square pockets were carried out with nominal constant width. The height of the two steps was instead varied in order to investigate the replication performance of the silicone when penetrating geometrical features with different depths. Moreover, the heights were set at a value that allows the micromilled pockets to be measured directly on the metal samples, thus making the comparison between original and replicated specimens feasible.

The width of the cavity was selected as reference for the replication assessment. Therefore, the dimensions *W1* and *W2* (see Figure 2) were measured on both metal master and replicated samples and then compared.

### 2.3. Replication Procedure

After the milling operation, the samples were cleaned from metal debris and dirty particles with ultrasonic cleaning and then blown with filtered air. The black two-component silicone rubber [19] was then poured on the steel masters by using the appropriate dispensing gun and following the supplier guidelines. The curing of the replication media was performed at room temperature. After complete solidification, the replicas were carefully removed from the master and prepared for the measurement, avoiding any additional contamination of the samples. In order to assess the repeatability of the replication, the procedure was repeated three times for each steel sample, resulting in three silicone replicas for each micromilled surface and pocket.

### 2.4. Surface Measurement Methodology and Uncertainty Evaluation Procedure

The surface topography measurements were carried out using a 3D confocal microscope (MarSurf CMW 100 from Mahr GmbH, Göttingen, Germany) with integrated white light interferometer employing a high-power 505 nm LED as light source. Both the measuring principles were employed in this study. Confocal microscopy is particularly suitable for 3D measurements of fine surfaces since it allows eliminating out-of-focus blurs by means of a selective pinhole [30]. The large numerical aperture of confocal microscopes objectives allows a very high maximum detectable slope (up to 75° [8]), which is particularly useful when dealing with the steep surface textures as those obtained by the micromilling process. Table 3 shows the main characteristics of the instrument.

**Figure 3:**
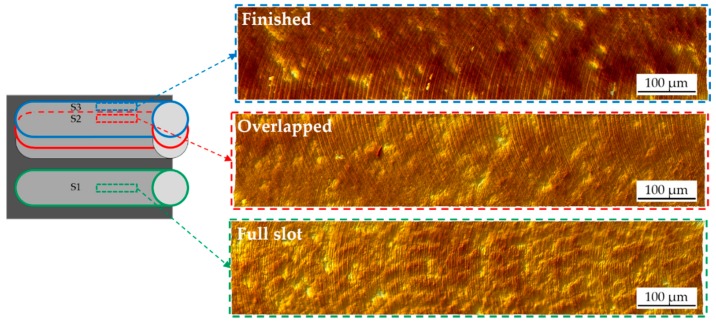
Surface texture appearance for the three acquired surfaces (AISI 440 H, Sample 2).

With the aim of characterizing a significant portion of the micromilled surface texture, a rectangular area of 1.0 mm × 0.2 mm was acquired for each steel and silicone surface. In particular, a stitching operation (with 9 images) was automatically performed for acquiring the entire surface extension. Such operation can be successfully applied to surface topography analysis, since it only has a minor influence on the results [31]. The acquisition of a relatively large area also decreases the effect of potential relocations errors on the comparison between masters and replicas. The position of S1, S2 and S3 was defined with respect to a fixed planar reference position univocally defined on both steel and silicone samples. The identification of such a reference is particularly important for comparing milled surfaces, which present a continuous surface texture. The raw surface acquisitions were post-processed with a dedicated image metrology software (MountainsMap^®^ [32], Digital Surf, Besançon, France). The images were initially flattened by means of a first order plane to ensure a correction for potential tilt of the samples. A peak-removal masking was also applied to remove a limited number of spikes that characterized the measured silicone surfaces. Due to their sharpness, these defects were considered as optical artifacts and therefore excluded from the quantitative texture analysis. Figure 3 shows the appearance of the three different micromilled surfaces. As expected, they present the typical characteristic of milled surfaces but with different patterns.

Finally, the performance verification of the replica technique was carried out by comparing masters and replicated surfaces using a synthetic surface parameter. In this case, the main objective was to assess the replication performance of the two-component silicone media with respect to the vertical profile development, since, given its nano-metric range, it is most critical to replicate. Therefore, the arithmetical mean height *Sa* was computed for each surface and then utilized as the parameter of comparison. This parameter, according to the ISO 25178-2 standard [33], gives indication about the average areal surface roughness of the surface, being the analogous of *Ra* that is used to describe profile measurements. *Sa* was chosen as indicator since it is only slightly influenced by local defects or optical artifacts, and therefore is suitable for a carrying out a general comparison between two surfaces. No cutoff filter was applied before calculating *Sa*. In this way, the performance replication throughout the entire spatial frequency domain was investigated.

To verify the quality of the measurements, an uncertainty evaluation was carried out. The measurement uncertainty *U* is a parameter associated with the results of a measurement that characterizes the dispersion of the values that could be reasonably be attributed to the measurand [34]. It is of paramount importance to determine this parameter and to include it in the evaluation of the replication capability, since, at the nano-scale, the variations due to replication process, instrument accuracy and measurement repeatability are in the same order of magnitude [4]. The uncertainty budget of the *Sa* measurements on the metal and silicone samples was estimated following ISO 15530-3 [35]. Although this method was conceived for CMM measurements, its principle can be successfully applied to optical measurements [23], allowing to avoid some of the complications that a full uncertainty estimation would introduce [36]. Such an approach is based on the substitution method, which allows estimating the instrument uncertainty by repeated measurements on a calibrated artifact sharing similar characteristic with the actual measurand. In this particular case, a calibrated roughness artifact (nominal value: *Ra* = 480 nm) was employed for this task. The measurement uncertainty related to surface roughness measurements when using the confocal microscope *u*_CONF_ was calculated as follows:

(1) $$u_{\mathrm{CONF}}=\sqrt{{u^{2}}_{\mathrm{cal}}+{u^{2}}_{p}+{u^{2}}_{\mathrm{res},\mathrm{CONF}}}$$

where *u*_cal_ is the standard calibration uncertainty of the roughness standard; *u*_p_ represents the standard uncertainty related to the measurement procedure and is calculated as standard deviation of fifteen repeated measurements on the calibrated standard; and *u*_res,CONF_ is the resolution uncertainty related to the declared 1 nm vertical resolution of the confocal microscope. The uncertainty contribution introduced by the thermal deformation of the samples was neglected since the measurements were performed inside a metrologic laboratory with a 20 °C ± 0.5 °C controlled temperature.

In order to evaluate the uncertainty related to the measurements of the actual measurands, one more source of uncertainty was taken into account: *u_Sa_*_, mill_, the standard deviation of ten repeated *Sa* measurements on the micromilled samples and on their replicas. Therefore, the expanded uncertainty of surface roughness measurement of the micromilled surfaces is calculated as:

(2) $$U_{\mathrm{Sa}}=k\text{×}\sqrt{{u^{2}}_{\mathrm{CONF}}+{u^{2}}_{\mathrm{Sa},\;\mathrm{mill}}}$$

where *k* is the coverage factor, equal to 2 for a 95% confidence interval. In order to characterize the uncertainty of both masters and replicas, *u_Sa_*_, mill_ was calculated for the three materials involved in the study. Table 4 presents the uncertainty budget.

The role of *U_Sa_* is fundamental in the replication performance verification: if the uncertainty intervals associated with direct and the indirect measurements overlap, the two outputs must be considered as equal. It is worth noting that the preponderant uncertainty contribution is represented by the calibration certificate of the roughness standard artifact.

### 2.5. Geometrical Measurement Methodology and Uncertainty Evaluation Procedure

The widths measurements of the micromilled pockets were carried out using a focus variation optical instrument (InfiniteFocus from Alicona Imaging GmbH, Raaba, Austria). This type of microscope is suitable for acquiring three-dimensional micro geometries but not for characterizing roughness of the order of tens of nanometers [37], which is typical of micromilled samples. Table 5 shows the main characteristics of this instrument.

**Figure 4:**
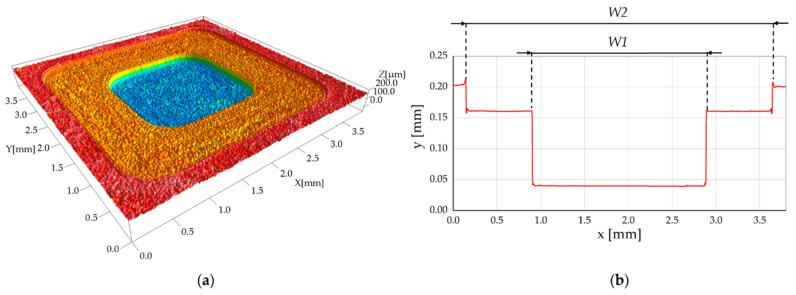
(**a**) Acquired 3D micromilled pocket (AISI 440 H, Pocket 3); and (**b**) extracted average profile and measurands *W1* and *W2*.

To evaluate the two measurands *W1* and *W2* (see Figure 2), the area corresponding to the whole pocket was acquired. Successively, *W1* and *W2* were extrapolated utilizing cross-sectional profiles (see Figure 4). In particular, one thousand parallel profiles were extrapolated and averaged and then the width was calculated as horizontal distance between the two points corresponding to the upper edge of the pocket vertical walls.

The uncertainty was evaluated using the same method applied for surface roughness measurements. In this case, a calibrated gauge block of 1.5 mm was selected as calibrated artifact. Therefore, the measurement uncertainty related to the width measurements using the focus variation instrument *u*_FV_ was calculated as:

(3) $$u_{\mathrm{FV}}=\sqrt{{u^{2}}_{\mathrm{cal}}+{u^{2}}_{p}+{u^{2}}_{\mathrm{res},\mathrm{FV}}}$$

where *u*_cal_ is the standard calibration uncertainty of the calibrated gauge and *u*_res,FV_ is the resolution uncertainty related to the 2.0 µm lateral resolution of the focus variation microscope.

As in the previous case, the final expanded uncertainty was determined by adding the contributions of the micromilled samples. Thus, the total uncertainty associated with the measurement of the pocket width was calculated as:

(4) $$U_{W}=k\times \sqrt{{u^{2}}_{\mathrm{FV}}+{u^{2}}_{W,\;\mathrm{mill}}}$$

where *k* is the coverage factor, equal to 2 for a 95% confidence interval and *u*_W, mill_ is the standard deviation of ten repeated width measurements on the micromilled metal and replicated samples. As before, this last contribution was determined for the three materials under investigation. As the standard deviation on *W1* and *W2* measurements was equal for each material, *U_W_* was applied to the measurements of both the measurands. Table 6 reports the resulting uncertainty budget.

## 3. Results and Discussion

### 3.1. Comparison of Surface Topography Measurements

**Figure 5:**
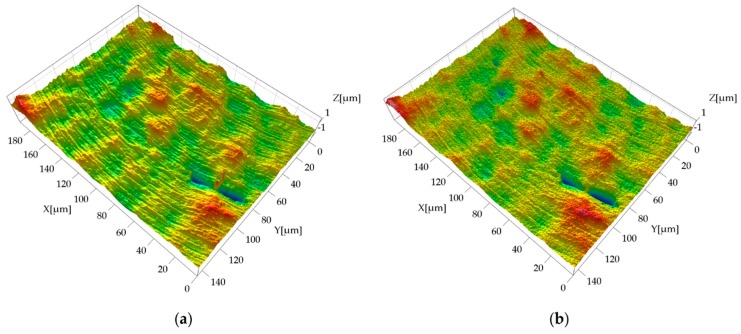
Detail of measured surface topography for AISI 440 H, surface S1, Sample 1: (**a**) metal master; and (**b**) silicone replica. The original silicone acquisition was inverted with respect to the *x-* and *z*-axes in order to facilitate the visual comparison. The *z*-axis was 20× magnified with respect to *x-* and *y*-axes.

The replicated surfaces generally showed a good resemblance with the masters. The surface marks created by the micro tool were reproduced with fidelity, even in their finest details (see Figure 5). Moreover, the silicone accurately reproduced local discontinuities in the surface texture.

**Figure 6:**
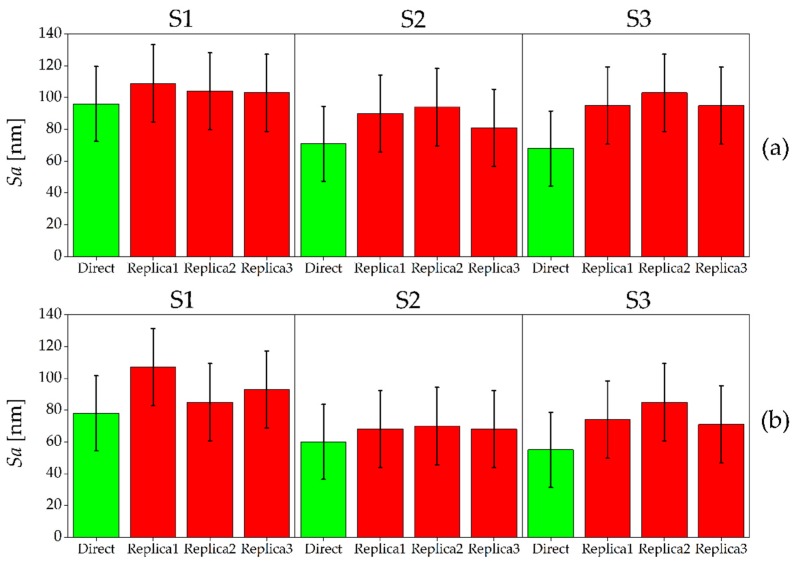
*Sa* values measured on master (green) and replicated (red) surfaces for AISI 440 H. The results for the three surfaces S1 (left), S2 (middle) and S3 (right) are shown: (**a**) Sample 1; and (**b**) Sample 2. The error bars indicate the expanded uncertainty *U_Sa_*.

The results of the quantitative surface characterization for the hardened mold steel are presented in Figure 6. For both Sample 1 and Sample 2, the direct measurement showed a decreasing average surface roughness when moving from the full slot surface (S1) to the other ones (S2 and S3). This is according to expectations, as S1 was machined in the cutting conditions that are typical of roughing operations. In general, *Sa* measured on the master surfaces ranged between 55 nm and 96 nm. A certain roughness variability was observed between the direct measurements of the two samples. In particular, Sample 1 presented an 11 nm to 18 nm larger *Sa* compared to Sample 2. This variability is due to the micromilling process itself, since the surfaces were machined in the same cutting conditions on the two samples. In comparison to the direct measurement, the three replicas always presented a larger *Sa*. However, considering the measurement uncertainty, direct and indirect measurements provided, in all the cases, the same *Sa* values. In fact, the uncertainty intervals overlap, making the measurement output equal from a metrological point of view. The standard deviations among the three silicone replicas range between 3 nm and 11 nm, demonstrating good repeatability of the replication procedure. Moreover, taking into account the measurement uncertainty, the replication procedure always provided consistent *Sa* results.

**Figure 7:**
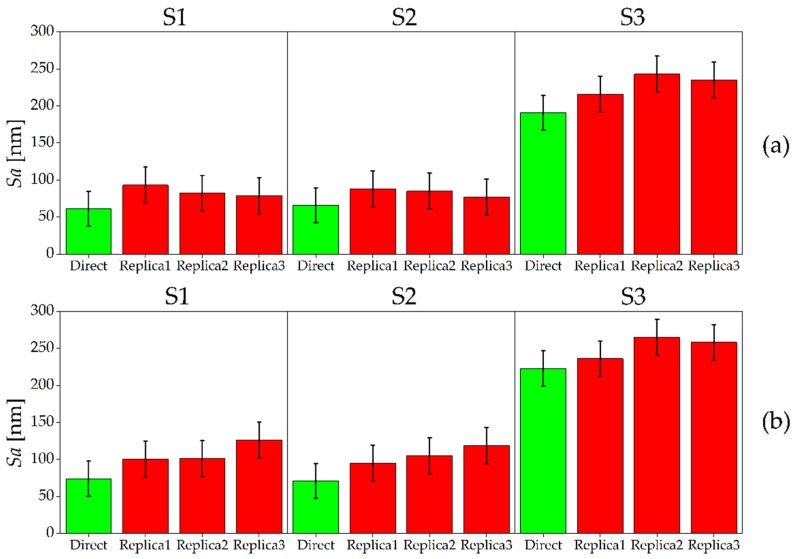
*Sa* values measured on master (green) and replicated (red) surfaces for AISI 440 A. The results for the three surfaces S1 (left), S2 (middle) and S3 (right) are shown: (**a**) Sample 1; and (**b**) Sample 2. The error bars indicate the expanded uncertainty *U_Sa_*.

**Figure 8:**
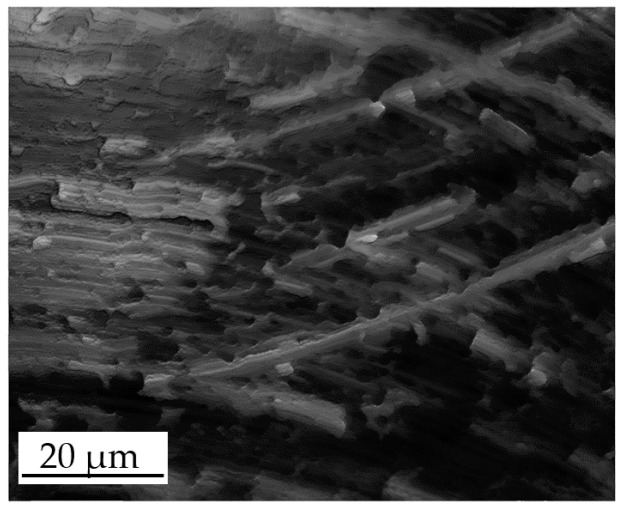
Detail of plowed marks on surface S3 of AISI 440 A material, Sample 2.

The results of the *Sa* measurements for the AISI 440 A are shown in Figure 7. In this case, S1 and S2 were produced with similar *Sa* roughness values (ranging between 61 nm and 74 nm for the two samples), while S3 had a significantly higher value of around 200 nm. This is contrary to expectations, as S3 was machined with the same radial depth of cut of S2. This unexpected increase of surface roughness was caused by the presence of plowing: for AISI 440 A, the last micromilling pass generated plowed marks on the surface, which diminished the surface quality (see Figure 8). This event happened only for S3 because the material accumulated on the tool during the first three passes (see Figure 1) leading to an increase of surface roughness [7] during the last pass. The softness of the annealed material is the most probable reason for this phenomenon. As for the previous material, a certain variability between Sample 1 and Sample 2 was observed. When comparing direct and indirect measurements, the replication process again introduced an average overestimation of the surface roughness of the master. However, as for the hardened material, the uncertainty intervals of direct and indirect measurements overlap in almost all cases, except for Replica 3 of S1, Sample 2 and Replica 2 of S3, Sample 1. The replication process is once again very repeatable: the three different replicas always provided the same measurements output, being the standard deviations among the three indirect measurements included between 7 nm and 15 nm.

**Figure 9:**
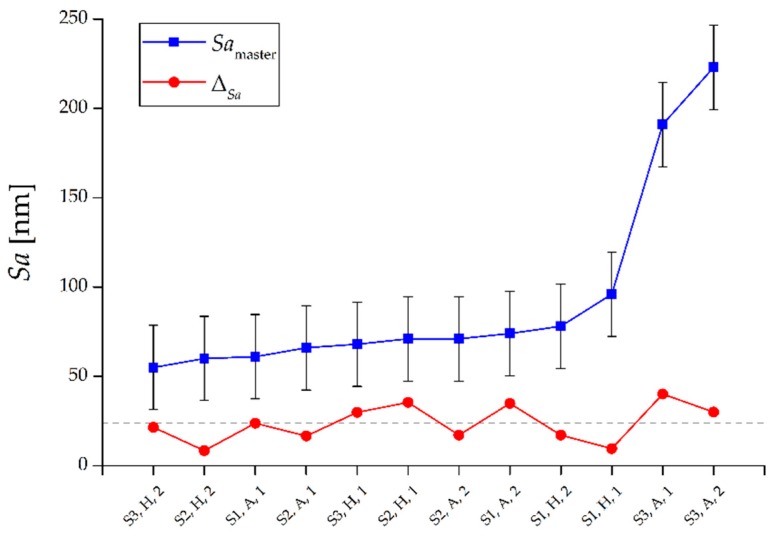
*Sa* values for the master surfaces and deviations ∆*_Sa_ = Sa*_replica_ − *Sa*_master_. The dashed grey line represents the average overestimation equal to 24 nm. The *x*-axis indicates the replicated surfaces by surface type (S1, S2 or S3), material (H or A) and Sample (1 or 2).

In both the hardened and annealed material, the replication procedure always introduced an overestimation of the roughness. The deviation ∆*_Sa_*, calculated as the difference between average *Sa* of the three replicas and *Sa* of the master, did not show any dependence on the master roughness (see Figure 9). In fact, while the roughness of metal surfaces increased, the deviation ∆*_Sa_* remained mostly constant around the value of 24 nm. The fact that the parameter ∆*_Sa_* assumed similar values for all the measured surfaces also demonstrates that there was not a dependence of the replication performance with respect to the two samples, the two materials and the three surface types, as also shown by the ANOVA results in Table 7. The p-values were in fact always much larger than 5% for both single factors and two-ways interactions.

To further investigate this experimental observation, other areal parameters were taken into account in order to understand which roughness component caused the *Sa* overestimation. In particular, the functional parameters *Svk*, *Spk* and *Sk* were calculated for masters and replicas and then compared. *Svk* is defined as the reduced dale height, and it provides an average indication of the valleys depth below the core roughness [33]. *Spk* is the reduced peak height and represents the mean height of peaks above the core surface [33]. Finally, *Sk* is used to characterize the core surface roughness [33]. In order to analyze how the silicone media replicated the valleys of the micromilled surfaces, it was chosen to compare the *Svk* parameter calculated on the masters with the *Spk* parameter of the corresponding replicas, since a valley on the master corresponds to a peak on the replica and vice versa. The core roughness was also utilized as parameter of comparison by means of *Sk* to determine whether the overestimation of the indirect *Sa* measurements is also related to an increase of this parameter.

**Figure 10:**
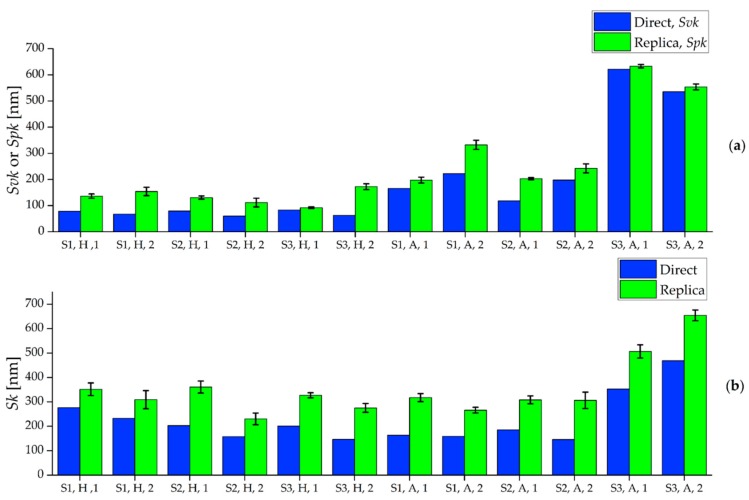
Functional roughness parameters calculated on master (blue) and replicated (green) samples: (**a**) *Svk* and *Spk* values for master and replicas respectively; and (**b**) *Sk* values. The interval bars indicate the standard deviations calculated among the three replicas. The *x*-axis indicates surface type (S1, S2 or S3), material (H or A) and Sample (1 or 2).

The results of the comparison are shown in Figure 10. It is possible to observe that the *Svk* values of the master are systematically lower than the *Spk* values of the replicated surfaces. In particular, the *Spk* of the replicas is on average 60% higher than the *Svk* of the masters. This clearly demonstrates that the replication procedure generated surfaces having peaks that are higher than the valleys of the master. The same happens for *Sk*: the indirect measurements provided an average 63% higher level of *Sk* for all the produced surfaces, indicating that the core roughness is also increased after the replication procedure. Therefore, the *Sa* overestimation introduced by the indirect measurements is caused by two distinct phenomena: the increase of the height of the peaks with respect to depths of the valleys and the increase of the core roughness.

This phenomenon is probably generated during the demolding phase of the replica. The two-component silicone during solidification penetrates in the master surface valleys, replicating the surface topography (see Figure 5). When the solidified replica is manually removed, the silicone sticks to the deepest points of the surface valleys due to their very small width, making its removal more difficult than in other areas. Therefore, it appears that manual removal causes a nano-metric stretch perpendicular to the average plane of the surface, which results in the observed increase of both dale height and core roughness. This also explains why the overestimation is almost constant (see Figure 9): the manual removal acts as external factor, and it does not depend on the experimental variables. Another evidence is given by the Kurtosis parameter *Sku* [33], which provides a quantitative evaluation of the sharpness of the roughness profile. The replicated surfaces had, on average, a 23% higher *Sku* value, demonstrating that they have a sharper profile than the masters, as a consequence of the stretch induced by the manual removal.

### 3.2. Comparison of Geometrical Measurements

**Figure 11:**
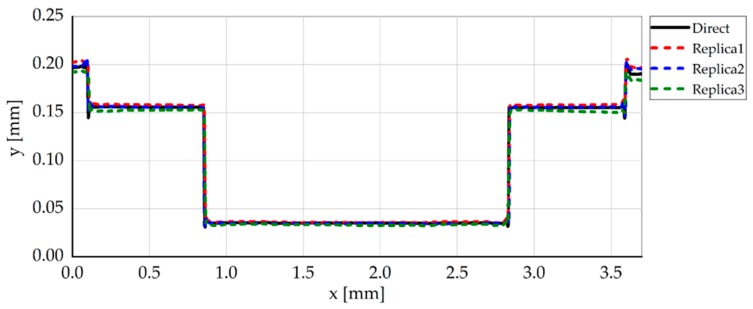
Extrapolated profiles for the direct and indirect width measurement. Original silicone acquisitions were inverted with respect to the *y*-axis in order to facilitate the visual comparison.

The steel micromilled pockets and their silicone replicas had a very similar shape. In particular, the replicas were able to accurately reproduce the geometry of the vertical walls, since no trace of any tilt due to the shrinkage of the polymer was present. With regard to the steps of the pockets, a not-perfect perpendicularity of the silicone pockets with respect to the vertical axis was sometimes observed, as shown in Figure 11 where Replica 3 presents a slightly tilted intermediate horizontal line. This micrometric deformation does not affect the width measurements.

**Figure 12:**
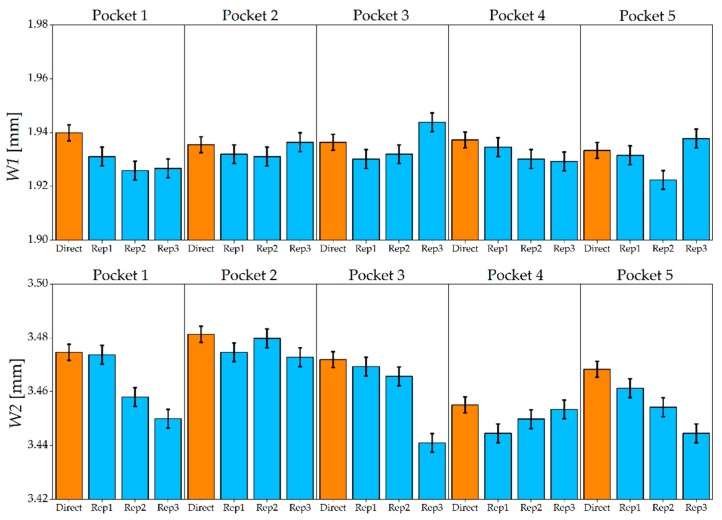
*W1* and *W2* measured on master (orange) and replicated (blue) pockets for AISI 440 H. The error bars indicate the expanded uncertainty *U_W_*.

Figure 12 shows the results of the width measurements made on the AISI 440 H sample. The five steel micromilled pockets have an almost constant *W1*, while a certain variability was observed for *W2*. When comparing master and replicated widths, it is possible to observe that the indirect measurement generally provided a lower value for both the measurands. In fact, only in 10% of the cases the indirect evaluation generated an overestimation of *W1* and *W2*. This finding in is accordance with the experiments carried out by Madsen et al. [21], in which a shrinkage between master and PDMS replica was observed when measuring lateral geometrical features. Direct and indirect measurements provided the same output (i.e., the uncertainty intervals overlapped) in 53% of the comparisons for *W1* and in 40% for *W2*, revealing that the replication fidelity was higher for the measurement of the inner geometry of the pockets (see Figure 2). As regards the repeatability of the replication procedure, the average standard deviation, calculated among the three replicas and for the five pockets, equals 5 µm for *W1* measurements and 9 µm for *W2* measurements. It is therefore possible to conclude that the *W1* was replicated more accurately and precisely by the silicone media.

**Figure 13:**
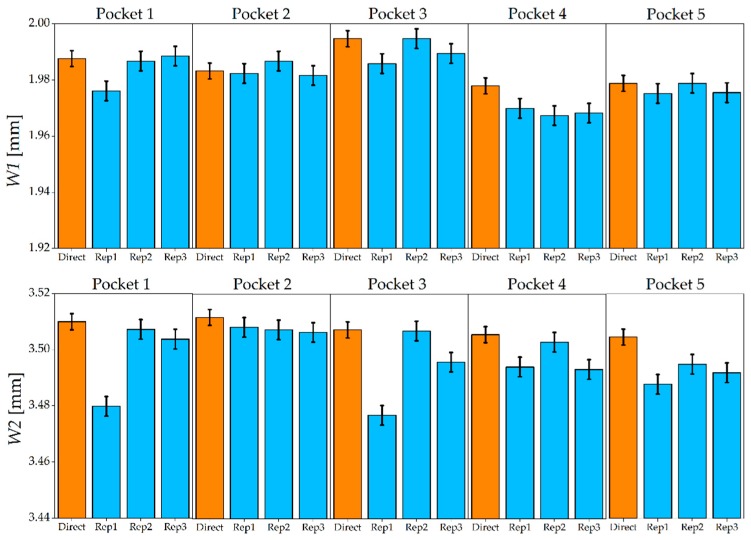
*W1* and *W2* measured on master (orange) and replicated (blue) pockets for the AISI 440 A. The error bars indicate the expanded uncertainty *U_W_*.

Figure 13 shows the results for the AISI 440 A sample. As in the harder material, the replication generally introduced an underestimation: only in one case out of 30 the width of the replicated specimen was larger than that of the master. The uncertainty intervals of direct and indirect measurements overlap in 67% of the cases for *W1* and in 47% of the cases for *W2*. Concerning the repeatability of the three replicas, average standard deviations of 3 µm and 8 µm were observed for *W1* and *W2*, respectively. Therefore, as for the hardened steel, a better replication was achieved for the internal width of the micromilled pockets.

**Figure 14:**
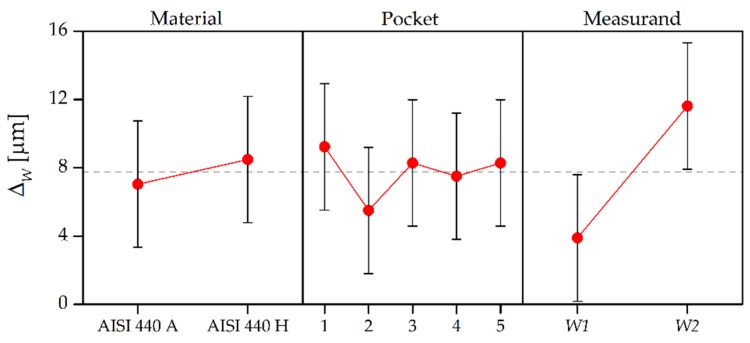
Main effects plot for the deviation ∆*_W_* = *W*_master_ − *W*_replica_. The error bars indicate the measurement uncertainty of ∆*_W_*, which was calculated applying the law of propagation of uncertainty [34].

The results in terms of deviation ∆*_W_*, calculated as the difference between direct and indirect measurement outputs, are summarized in Figure 14 and Table 8 in which the ANOVA results for ∆*_W_* are shown. It may be seen that average deviation was approximately 8 µm, demonstrating that the silicone media was able to achieve replication fidelity down to a single micrometer digit. The replication performance did not depend on the material of the master, as the p-value was larger than 5% for this experimental factor. The same conclusion can be drawn for the five pockets: the replication performance was unaffected by the different depth of the geometry under indirect measurement. On the contrary, the fidelity of the replicas was greatly affected by the type of measurand. When measuring *W2*, the deviation was on average 8 µm higher than when measuring *W1*. This suggests that the silicone media better replicates geometries that are more internal with respect to the outer surface on which it is applied (see Figure 2).

In order to further characterize the behavior of the silicone when replicating geometrical features, the shrinkage *s_W_* was calculated as:

(5) $$s_{W}=\frac{W_{\mathrm{master}}-W_{\mathrm{replica}}}{W_{\mathrm{master}}}\;\%$$

where *W*_master_ and *W*_replica_ are the width measurements carried out on the master and on the replica, respectively. By considering both *W1* and *W2*, the average shrinkage equaled 0.27% ± 0.03%, where the last value represents the expanded uncertainty calculated by means of the law of propagation of error [34] applied to Equation (5). This parameter is particularly useful, since it gives precise indications on what is the percentage underestimation introduced by the replication procedure when measuring micromilled geometries. Therefore, it can be used to determine the dimension of the master when only an indirect measurement is available, provided that the uncertainty value of the shrinkage is taken into account.

## 4. Conclusions

The present paper investigated the replication performance of a commercial silicone replica media applied as an indirect measurement tool for micromilled components. The replication capabilities related to surface and geometrical characterization were assessed separately by using two specifically designed micromilled samples. An uncertainty evaluation procedure based on ISO 15530-3 was used to compare the results provided by direct and indirect measurements.

The analysis of surface roughness, based on the three-dimensional areal parameter *Sa*, was carried out using a confocal microscope. Three different types of surface were machined on two mold steels and successively replicated. A qualitative comparison between masters and their replicas showed that the replication media was capable of accurately reproducing the appearance of the micromilled surface texture. A numerical comparison revealed that the indirect measurements always overestimated the average roughness of the master. By taking into account functional parameters such as *Svk*, *Spk*, and *Sk*, it was demonstrated that the overestimation introduced by the replication procedure is due to an increase of both core roughness and average peak height. The *Sa* deviation assumed an average value of 24 nm and was unaffected by the experimental variables (i.e., surface type and material). This strongly suggests that such effect was caused by an external factor such as the manual detachment of the silicone replicas from the master surface. However, considering the measurement uncertainty, the indirect and direct measurements provided the same results in 34 cases out of 36, demonstrating that the replication media used was suitable for characterizing micromilled surfaces that are inaccessible for other measurement systems.

The investigation related to the geometry replication focused on the measurement of widths of micromilled pockets featuring a two-stepped profile. Measurements were performed by means of a focus variation optical instrument. The tests were carried out on the same two materials employed in the previous analysis. Results showed that the replicated geometries were generally smaller than the metal masters. This underestimation was related to the type of measurand: for more internal geometries, such as the lower step of the pockets, the error was smaller, while it was larger for geometries that were more exposed to the pouring of the replication media. In view of the calculated expanded uncertainty, the direct and indirect width measurements provided the same result in 31 cases out of 60, demonstrating that the replication performance did not allow meeting the target consistently. However, considering the average shrinkage equal to 0.27% ± 0.03%, the master dimensions and the associated measurement uncertainty can be derived from indirect measurements performed with the investigated silicone media.

Future research will be dedicated to the study of different types of replication media and to micromilled components presenting more complex and free-form shapes.

## Figures and Tables

**Table 1:** Cutting parameters for the three machined surfaces.

Cutting Parameter	S1	S2	S3
*a*_e_/*D*_C_	100% (full slot)	30% (overlapped)	30% (finished)
*a*_p_/µm	50	50	50
*v*_c_/(m/min)	100	100	100
*f*_z_/µm	12.5	12.5	12.5

**Table 2:** Cutting parameters for the five machined pockets.

Cutting Parameter	Value
*a*_e_/mm	200
*a*_p_/µm	150
*v*_c_/(m/min)	60
*f*_z_/µm	10

**Table 3:** Confocal microscope characteristics.

Objective Magnification	100×
Numerical aperture	0.90
Working distance in mm	1.0
Field of view in µm	192 × 144
Optical lateral resolution in µm	0.28
Digital lateral resolution in µm	0.25
Vertical resolution in nm	1.0

**Table 4:** Uncertainty contributions for the *Sa* roughness measurements on the masters and replicated surfaces.

Uncertainty Contribution	AISI 440 H	AISI 440 A	Silicone
*u*_cal_/nm	11.0	11.0	11.0
*u*_p_/nm	2.1	2.1	2.1
*u*_res,CONF_/nm	0.3	0.3	0.3
*u*_CONF_/nm	11.2	11.2	11.2
*u_Sa_*_, mill_/nm	3.7	3.9	4.7
*U_Sa_*/nm	24	24	24

**Table 5:** Focus variation instrument characteristics.

Objective Magnification	10×
Numerical aperture	0.30
Working distance in mm	16.0
Field of view in µm	1429 × 1088
Digital lateral resolution in µm	0.88
Vertical resolution in nm	200.0

**Table 6:** Uncertainty contributions for *W1* and *W2* measurements on the masters and replicated samples.

Uncertainty Contribution	AISI 440 H	AISI 440 A	Silicone
*u*_cal_/µm	0.5	0.5	0.5
*u*_p_/µm	0.7	0.7	0.7
*u*_res,FV_/µm	0.6	0.6	0.6
*u*_FV_/µm	1.0	1.0	1.0
*u_W_*_, mill_/µm	1.2	1.1	1.5
*U_W_*/µm	3.1	3.0	3.6

**Table 7:** ANOVA table for deviations ∆*_Sa_* between replicated and master *Sa* surface roughness.

Factor	Adj. MS	F-Value	*p*-Value
Material	136.5	2.2	0.27
Surface type	137.7	2.3	0.31
Sample	57.7	1.0	0.43
Material × Surface type	115.8	1.9	0.34
Material × Sample	71.2	1.2	0.39
Surface type × Sample	145.2	2.4	0.30
Error	60.8		

**Table 8:** ANOVA table for deviations ∆*_W_* between direct and indirect measurement of *W1* and *W2*.

Factor	Adj. MS	F-Value	*p*-Value
Material	32.2	0.6	0.46
Pocket	42.2	0.8	0.57
Measurand	800.2	14.1	0.00
Material × Pocket	56.9	1.0	0.42
Material × Measurand	5.6	0.1	0.76
Pocket × Measurand	69.8	1.2	0.31
Error	56.7		

## References

[1] Alting L., Kimura F., Hansen H.N., Bissacco G. (2003). Micro Engineering. CIRP Ann..

[2] Brousseau E.B., Dimov S.S., Pham D.T. (2010). Some recent advances in multi-material micro- and nano-manufacturing. Int. J. Adv. Manuf. Technol..

[3] Bissacco G., Hansen H.N., De Chiffre L. (2005). Micromilling of hardened tool steel for mould making applications. J. Mater. Process. Technol..

[4] Hansen H.N., Hocken R.J., Tosello G. (2011). Replication of micro and nano surface geometries. CIRP Ann. Manuf. Technol..

[5] Parenti P., Masato D., Sorgato M., Lucchetta G., Annoni M. (2017). Surface footprint in molds micromilling and effect on part demoldability in micro injection molding. J. Manuf. Process..

[6] Liu X., DeVor R.E., Kapoor S.G., Ehmann K.F. (2004). The Mechanics of Machining at the Microscale: Assessment of the Current State of the Science. J. Manuf. Sci. Eng..

[7] Weule H., Hüntrup V., Tritschler H. (2001). Micro-Cutting of Steel to Meet New Requirements in Miniaturization. CIRP Ann. Manuf. Technol..

[8] Hansen H.N., Carneiro K., Haitjema H., De Chiffre L. (2006). Dimensional micro and nano metrology. CIRP Ann. Manuf. Technol..

[9] Hocken R.J., Chakraborty N., Brown C. (2005). Optical Metrology of Surfaces. CIRP Ann. Manuf. Technol..

[10] Leach R.K. (2011). Optical Measurement of Surface Topography.

[11] Yao D., Kim B. (2002). Injection molding high aspect ratio microfeatures. J. Inject. Molding Technol..

[12] Mcfarland A.W., Poggi M.A., Bottomley L.A., Colton J.S. (2004). Injection moulding of high aspect ratio micron-scale thickness polymeric microcantilevers. Nanotechnology.

[13] Liou A.C., Chen R.H. (2006). Injection molding of polymer micro- and sub-micron structures with high-aspect ratios. Int. J. Adv. Manuf. Technol..

[14] Bachmann W., Jean B., Bende T., Wohlrab M., Thiel H.J. (1993). Silicone replica technique and automatic confocal topometry for determination of corneal surface roughness. Ger. J. Ophthalmol..

[15] Scott R.S., Ungar P.S., Bergstrom T.S., Brown C.A., Childs B., Teaford M.F., Walker A. (2006). Dental microwear texture analysis. J. Hum. Evol..

[16] Zuljan D., Grum J. Non-destructive metallographic analysis of surfaces and microstructures by means of replicas. Proceedings of the 8th International Conference of the Slovenian Society Non-Destructive Testing, Application of Contemporary Non-Destructive Testing in Engineering.

[17] Jordon J.B., Bernard J.D., Newman J.C. (2012). Quantifying microstructurally small fatigue crack growth in an aluminum alloy using a silicon-rubber replica method. Int. J. Fatigue.

[18] AccuTrans 2012 AccuTrans Brochure. http://www.accutrans.info/fileadmin/dam/DATEN/AccuTrans/downloads/others/30000992_11–12_IFU_AccuTrans_AM_01.pdf.

[19] Struers 2008 RepliSet Brochure. http://www.struers.com/en-GB/Products/Materialographic-analysis/Materialographic-analysis-equipment/Replication-system.

[20] Tosello G., Hansen H.N., Gasparin S., Albajez J.A., Esmoris J.I. (2012). Surface wear of TiN coated nickel tool during the injection moulding of polymer micro Fresnel lenses. CIRP Ann. Manuf. Technol..

[21] Madsen M.H., Feidenhans’l N.A., Hansen P.-E., Garnæs J., Dirscherl K. (2014). Accounting for PDMS shrinkage when replicating structures. J. Micromech. Microeng..

[22] Goodall R.H., Darras L.P., Purnell M.A. (2015). Accuracy and precision of silicon based impression media for quantitative areal texture analysis. Sci. Rep..

[23] Gasparin S., Hansen H.N., Tosello G. Traceable surface characterization using replica moulding technology. Proceedings of the 13th International Conference on Metrology Properties of English Surfaces, Kgs.

[24] Jinsheng W., Dajian Z., Yadong G. (2009). A Micromilling Experimental Study on AISI 4340 Steel. Key Eng. Mater..

[25] Kiswanto G., Zariatin D.L., Ko T.J. (2014). The effect of spindle speed, feed-rate and machining time to the surface roughness and burr formation of Aluminum Alloy 1100 in micro-milling operation. J. Manuf. Process..

[26] Wang J., Gong Y., Shi J., Abba G. Surface Roughness Prediction in Micromilling using Neural Networks and Taguchi’s Design of Experiments. Proceedings of the IEEE International Conference on Industrial Engineering and Engineering Managament (IEEM).

[27] Cardoso P., Davim J.P. (2010). Optimization of Surface Roughness in Micromilling. Mater. Manuf. Process..

[28] Liu X., DeVor R.E., Kapoor S.G. (2007). Model-Based Analysis of the Surface Generation in Microendmilling—Part I: Model Development. J. Manuf. Sci. Eng..

[29] Abdelrahman Elkaseer A.M., Dimov S.S., Popov K.B., Negm M., Minev R. (2012). Modeling the Material Microstructure Effects on the Surface Generation Process in Microendmilling of Dual-Phase Materials. J. Manuf. Sci. Eng..

[30] Schwenke H., Neuschaefer-Rube U., Pfeifer T., Kunzmann H. (2002). Optical Methods for Dimensional Metrology in Production Engineering. CIRP Ann. Manuf. Technol..

[31] Marinello F., Bariani P., De Chiffre L., Hansen H.N. (2007). Development and analysis of a software tool for stitching three-dimensional surface topography data sets. Meas. Sci. Technol..

[32] MountainsMap^®^, Digital Surf. http://www.digitalsurf.fr/en/mntkey.html.

[33] ISO (2012). IOS 25178-2: Geometrical Product Specifications (GPS)—Surface Texture: Areal—Part 2: Terms, Definitions and Surface Texture Parameters.

[34] Joint Committee for Guides in Metrology (JCGM) (2008). Evaluation of Measurement Data: Guide to the Expression of Uncertainty in Measurement.

[35] ISO (2011). IOS 15530-3: Geometrical Product Specifications (GPS)—Coordinate Measuring Machines (CMM): Technique for Determining the Uncertainty of Measurement.

[36] Haitjema H. (2015). Uncertainty in measurement of surface topography. Surf. Topogr. Metrol. Prop..

[37] Tosello G., Haitjema H., Leach R.K., Quagliotti D., Gasparin S., Hansen H.N. (2016). An international comparison of surface texture parameters quantification on polymer artefacts using optical instruments. CIRP Ann. Manuf. Technol..

